# Comparison between Exhaustive and Equilibrium Extraction Using Different SPE Sorbents and Sol-Gel Carbowax 20M Coated FPSE Media

**DOI:** 10.3390/molecules24030382

**Published:** 2019-01-22

**Authors:** Angela Tartaglia, Marcello Locatelli, Abuzar Kabir, Kenneth G. Furton, Daniela Macerola, Elena Sperandio, Silvia Piccolantonio, Halil I. Ulusoy, Fabio Maroni, Pantaleone Bruni, Fausto Croce, Victoria F. Samanidou

**Affiliations:** 1 Department of Pharmacy, University of Chieti–Pescara “G. d’Annunzio”, Via dei Vestini 31, 66100 Chieti, Italy; angela.tartaglia@unich.it (A.T.); daniela.macer@gmail.com (D.M.); sperandioelena94@gmail.com (E.S.); silvia.piccolantonio@studenti.unich.it (S.P.); fabio.maroni@unich.it (F.M.); pantaleonebruni@libero.it (P.B.); fausto.croce@unich.it (F.C.); 2 Department of Chemistry and Biochemistry, International Forensic Research Institute, Florida International University, 11200 SW 8th St, Miami, FL 33199, USA; furtonk@fiu.edu; 3 Department of Analytical Chemistry, Faculty of Pharmacy, Cumhuriyet University, Sivas 58140, Turkey; hiulusoy@yahoo.com; 4 Laboratory of Analytical Chemistry, Department of Chemistry, Aristotle University of Thessaloniki, 54124 Thessaloniki, Greece; samanidu@chem.auth.gr

**Keywords:** FPSE, in-house loaded SPE, HPLC-PDA, method validation, IBD, extraction

## Abstract

This paper reports the performance comparison between the exhaustive and equilibrium extraction using classical Avantor C18 solid phase extraction (SPE) sorbent, hydrophilic-lipophilic balance (HLB) SPE sorbent, Sep-Pak C18 SPE sorbent, novel sol-gel Carbowax 20M (sol-gel CW 20M) SPE sorbent, and sol-gel CW 20M coated fabric phase sorptive extraction (FPSE) media for the simultaneous extraction and analysis of three inflammatory bowel disease (IBD) drugs that possess logP values (polarity) ranging from 1.66 for cortisone, 2.30 for ciprofloxacin, and 2.92 for sulfasalazine. Both the commercial SPE phases and in-house synthesized sol-gel CW 20M SPE phases were loaded in SPE cartridges and the extractions were carried out under an exhaustive extraction mode. FPSE was carried out under an equilibrium extraction mode. The drug compounds were resolved using a Luna C18 column (250 mm × 4.6 mm; 5 μm particle size) in gradient elution mode within 20 min and the method was validated in compliance with international guidelines for the bioanalytical method validation. Novel in-house synthesized and loaded sol-gel CW 20M SPE sorbent cartridges were characterized in terms of their extraction capability, breakthrough volume, retention volume, hold-up volume, number of the theoretical plate, and the retention factor.

## 1. Introduction

Sample preparation and ‘clean-up’ have the aim of improving the analytical parameters to enhance detectability and to protect the performance of analytical equipment. However, these steps are time consuming, utilize large volumes of toxic and hazardous organic solvents, and are prone to error. For these reasons, novel extraction technologies are continuously being developed to overcome these drawbacks [1] in order to improve the extraction efficiency, reduce the sample handling, and enhance the reproducibility.

Novel extraction strategies focus on improving the quality of extraction, decreasing the use of solvents to become environmentally friendly, reducing the time for extraction, and the associated costs. Solid-phase extraction (SPE) is the most popular sample preparation technique used for extraction, clean up, and pre-concentration of both environmental and biological samples. In addition, SPE is fast, easy, and green due to its reduced consumption of solvent compared to liquid–liquid extraction, its relative inexpensiveness and ability for easy automation as well as shorter analysis time. The primary advantage is that SPE is a non-equilibrium, exhaustive extraction procedure; the problem with using an equilibrium process is that the analyst may never know when equilibrium has been reached, and the equilibrium distribution may necessitate multiple extractions [2].

Fabric phase sorptive extraction (FPSE) is a new generation solvent-free/solvent-minimized microextraction technique developed by Kabir and Furton [3]. FPSE is a simple, rapid, and green sample preparation technique, which not only simplifies the sample preparation by eliminating many unnecessary steps such as filtration, protein precipitation, solvent evaporation, and sample reconstitution, it also substantially reduces the organic solvent consumption [4]. The permeable fabric substrate used to chemically bind sol-gel derived high performance sorbents in FPSE mimics the flow-through extraction mechanism used in SPE even when the extraction is carried out under equilibrium extraction mode, resulting in a faster mass transfer and extraordinarily high analyte recovery. The flexibility of the FPSE media allows its direct insertion into the sample container, reducing possible analyte loss [1] and potential cross contamination stems from the containers. The main advantages of FPSE are its high contact surface area for rapid sorbent-analyte interactions and the possibility of enhancing the diffusion of analytes through the FPSE media with magnetic stirring or sonication to obtain a fast extraction equilibrium [5].

Among different treatment regimens used in inflammatory bowel disease (IBD), cortisone, ciprofloxacin, and/or sulfasalazine are orally administrated among other drugs; these drugs are generally present at trace level concentrations in physiological samples including plasma, urine, and saliva. As such, a preconcentration step is necessary to obtain advantageous signal-to-noise ratios in chromatographic separation and detection [6,7]. The logP values of the target analytes ranging from 1.66 for cortisone, to 2.30 for ciprofloxacin, and 2.92 for sulfasalazine (Table 1), coupled to their widespread range of pK_a_ values pose a great challenge for their simultaneous extraction and analysis. Particularly these differences could influence the extraction efficiency of the analytes. These differences necessarily guide the selection of possible sample preparation and clean-up procedures that can be applied, and failure to select an appropriate sample preparation technique may lead to low analytical performances during traces analysis.

Both the exhaustive extraction technique (such as solid phase extraction) and equilibrium extraction technique (such as solid phase microextraction, fabric phase sorptive extraction, stir bar sorptive extraction) not only utilize different extraction mechanism, but also use mutually exclusive extraction sorbents. Among SPE sorbents, hydrophilic-lipophilic balance (HLB) and C18 are the most popular, whereas for equilibrium extraction, poly(dimethyl siloxane), poly(ethylene glycol), and polyacrylate are noteworthy. As such, it is important to compare the extraction performances between the two major extraction modes as well as between different sorbent chemistries. Only a few sorbent chemistries are available in both the exhaustive and equilibrium extraction formats. However, our research group has recently introduced a large number of traditional microextraction sorbents including poly(dimethyl siloxane) and poly(ethylene glycol) in solid phase extraction format. In the current study, sol-gel CW 20M SPE sorbent and sol-gel CW 20M coated FPSE media are used for extraction performance evaluation and comparison among other sorbents. These phases were newly synthesized in our laboratory and herein tested in order to compare them in terms of the main analytical parameters (for SPE breakthrough volume (*V_B_*), retention volume (*V_R_*), hold-up volume (*V_M_*), retention factor (*k*), and theoretical plates number (*N*), and for both SPE and FPSE the enrichment factors).

To the best of our knowledge, this is the first comprehensive comparison between exhaustive and equilibrium extraction modes utilizing multiple C18 SPE sorbents, HLB sorbent, novel sol-gel CW 20M SPE sorbent, and sol-gel CW 20M coated FPSE membrane for the determination of IBD drugs, possessing logP values (polarity) of 1.66 for cortisone, 2.30 for ciprofloxacin, and 2.92 for sulfasalazine. Due to the difference in their physicochemical properties, especially their polarity, selected IDB drugs pose a challenge for their simultaneous extraction and preconcentration at their trace and ultra-trace level concentrations.

## 2. Results and Discussion

### 2.1. Determination of SPE Parameters: Breakthrough Volume, Retention Volume, Hold-Up Volume, Retention Factor, and Theoretical Plate Number for SPE Devices

To understand the extraction mechanism and to utilize their full potential as the extraction media, it is important to characterize the sorbents available for the extraction and clean-up process. When the solid phase extraction (SPE) procedure is adopted, the main parameters that need to be considered are the breakthrough volume (*V_B_*), retention volume (*V_R_*), hold-up volume (*V_M_*), retention factor (*k*), and the theoretical plates number (*N*).

As reported in the literature [8,9], for the determination of the above parameters, *off-line* SPE can be followed by applying the sigmoid shape breakthrough curve. Particularly, from this model obtained by fitting of the experimental points by Boltzmann’s function:

(1) $$Y=A_{2}+\frac{A_{1}-A_{2}}{1+e^{\frac{x-x_{0}}{dx}}}$$

and be calculated by the breakthrough volume

(2) $$V_{B}=x_{0}+dx\cdot ln\left[\frac{100}{99}\left(1-\frac{A_{1}}{A_{2}}\right)-1\right]$$

hold-up volume:

(3) $$V_{M}=x_{0}+dx\cdot ln\left[99-100\cdot \frac{A_{1}}{A_{2}}\right]$$

The retention volume, defined as the inflection point of the curve, corresponds to the *x*_0_ value. The retention factor (*k*) is defined using the following equation:

(4) $$V_{M}=V_{R}\left(1+k\right)$$

and the theoretical plates number:

(5) $$N=\frac{V_{R}\left(V_{R}-\sigma_{V}\right)}{\sigma_{V}^{2}}$$

were $\sigma_{V}$ was obtained by:

(6) $$V_{B}=V_{R}-2\sigma_{V}$$

In the current study, following our research on innovative extraction procedures [4,10,11,12,13] and devices, we compare four different SPE sorbents (both commercially available and in-house produced) and the innovative fabric phase sorptive extraction (FPSE) media.

**Figure 1:**
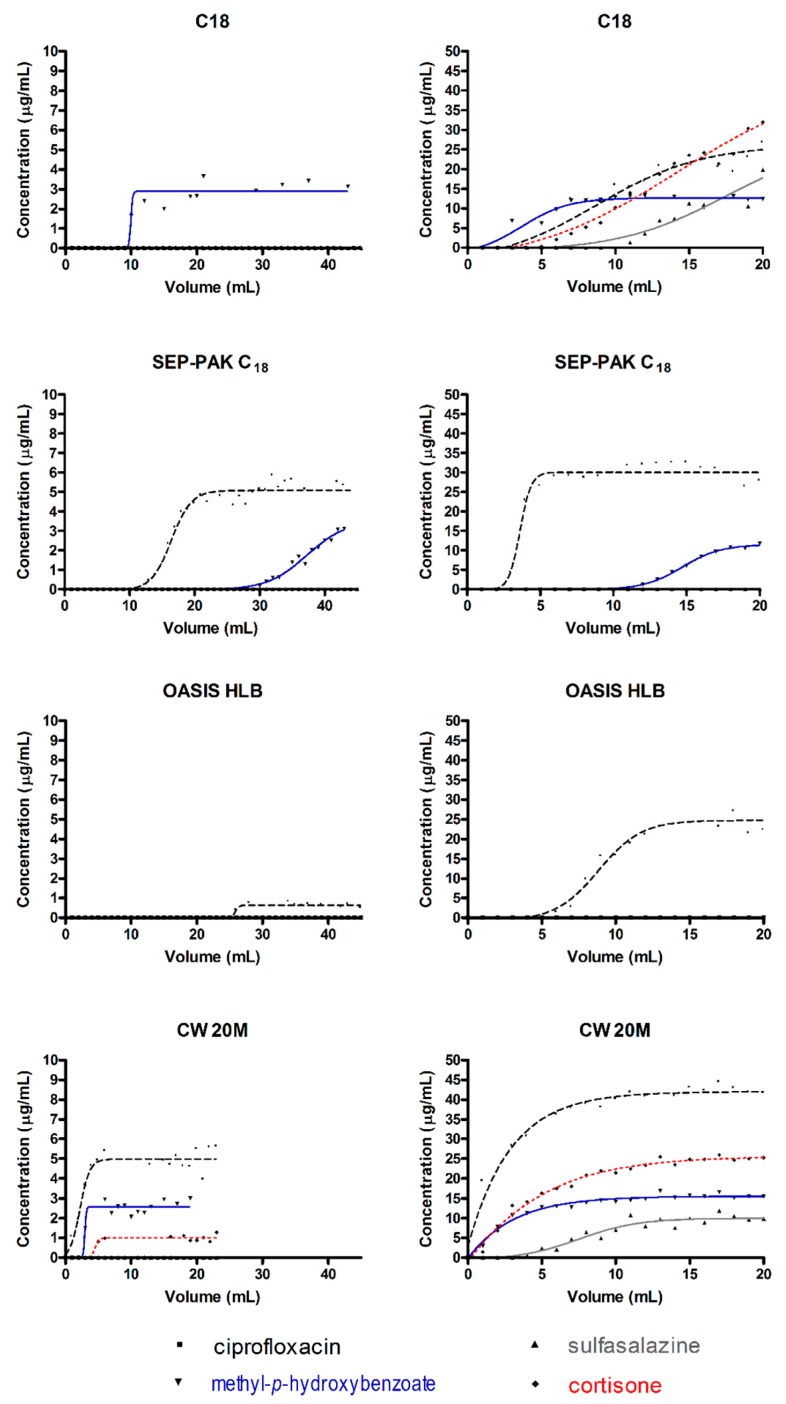
Comparison of breakthrough curves determined for the analytes on the different solid phase extraction (SPE) sorbents and at 10 μg/mL (**left**) and 50 μg/mL (**right**).

Using these equations, it was possible to evaluate the breakthrough volume, retention volume, hold-up volume, retention factor, and theoretical plate number for the herein considered stationary phases loaded on the SPE cartridges. In Figure 1 we reported the experimental curves and the fittings used for the parameters calculation.

The Boltzmann’s functions shown in Figure 1 allows us to evaluate the breakthrough volume (*V_B_*), retention volume (*V_R_*), hold-up volume (*V_M_*), retention factor (*k*), and theoretical plate number (*N*) as reported in Table 2 and Table 3 (by using the above reported equations). In these Tables, it was highlighted that for the tested compounds, some of these materials are not available in a “universal” phase. In fact, some analytes were not reported (both in Figure 1 and Table 2 and Table 3) because no quantitative data were obtained after the SPE steps and, consequently, it was not possible to perform the analysis by Boltzmann’s function (like all the other compounds). Similarly, in other published papers [8,9,14], this model could not reach a complete analysis (due to a “floating point error” applying Boltzmann’s function) and evaluation of the above-mentioned parameters.

All tested SPEs show high retention values for sulfasalazine that do not allow us to evaluate the principal parameters, also due to the fact that with the obtained quantitative data no Boltzmann’s function can be performed. Within the four tested phases, the sol-gel CW 20M shows the ability to retain all compounds with a good number of theoretical plates, and in the meantime allow the use of a small elution volume. These findings follow the general principles of the Green Analytical Chemistry. In this case, unfortunately, the calculated breakthrough volume, retention volume, hold-up volume, retention factor, and theoretical plate number were lower than the values obtained for the other tested phases.

Interestingly, commercially available phases show superior results at higher concentration levels for the tested compounds, while the new sol-gel CW 20M are at low concentrations. This finding could be observed from the comparison of Table 2 and Table 3 where the evaluated parameters can be calculated for high and low concentrations, respectively.

### 2.2. Mechanism of Extraction in FPSE

FPSE has combined two major sample preparation techniques: SPE (governed by exhaustive extraction principle) and SPME (governed by equilibrium driven extraction principle) into a single sample preparation technique. Due to the geometrical advantage and the combination of both the equilibrium and exhaustive extraction mechanism [15], FPSE can perform exhaustive extraction under equilibrium extraction conditions. The fabric substrates are inherently permeable. The permeability of the fabric substrate remains intact even after the sol-gel sorbent coating. When the FPSE media is immersed into the liquid sample matrix, the aqueous solution rapidly permeates through the porous bed of sol-gel sorbent-coated FPSE media and rapidly interacts with the sorbent via different intermolecular interaction mechanisms. As such, the FPSE membrane behaves like a SPE disk. At the same time, when the FPSE media is introduced into the aqueous solution, it behaves similarly to the SPME thin film format, analytes continue to accumulate onto the FPSE media based on the partition coefficient of the analyte(s) between the sol-gel sorbent and the sample matrix until the extraction equilibrium is reached. 

### 2.3. Characterization of Sol-Gel CW 20M SPE Sorbent Using FTIR and TGA Analysis

**Figure 2:**
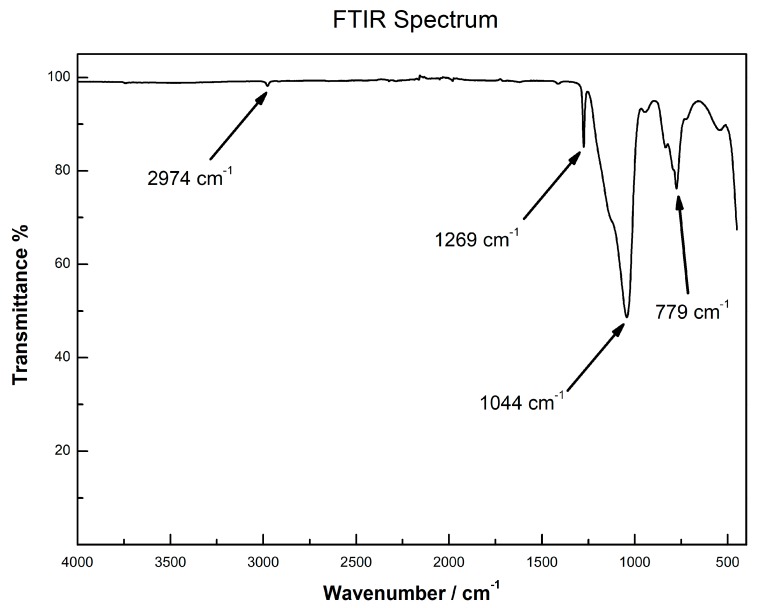
FTIR spectrum for the CW 20M stationary phase.

The experimental FTIR spectrum shows a very weak band at 2974 cm^−1^, indicating the symmetric stretching of a C-H bond. Then a medium sharp band a 1269 cm^−1^, which can be assigned to a Si-CH_3_ bond and is indicative of the successful integration of methyl trimethoxysilane (MTMS) into the sol-gel network. Then a broad band with a visible shoulder, centered at 1044 cm^−1^, indicative of vibrations related to Si-O-Si bonds. These features are likely connected to the siloxane nature of the sample [16]. All these findings are reported in Figure 2. One very important point that can be noted here, is the absence of any band around 3690 cm^−1^ that corresponds to free silanol (Si-OH). This indicates that even though the sol-gel CW 20M SPE sorbent was not treated with any endcapping (post synthesis derivatization to minimize unreacted surface silanol groups of the silica substrate), the novel SPE sorbent does not possess any residual surface silanol groups.

**Figure 3:**
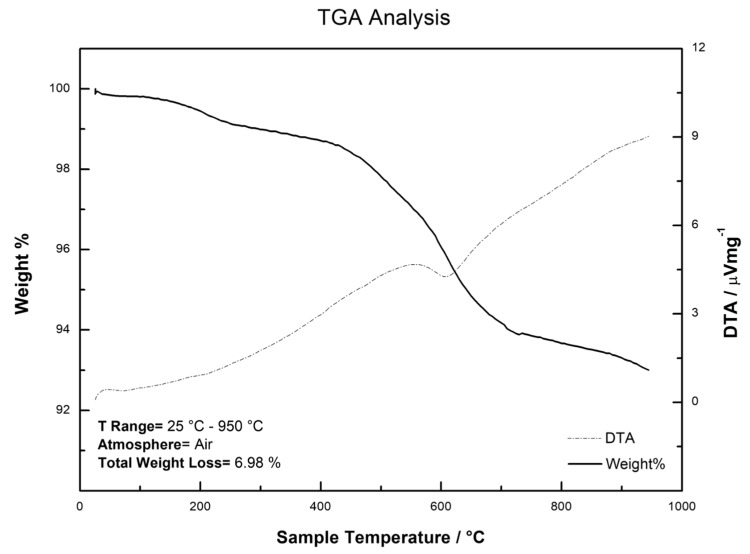
TGA analysis for the CW 20M stationary phase.

Thermogravimetric analysis shows a 2% weight loss up to 250 °C, probably due to loss of moisture and volatile species entrapped inside the sol-gel CW20M particles. Then, as indicated by differential thermal analysis (DTA) data, between 400 °C and 600 °C a slightly exothermic process is visible, probably due to the loss of unreacted reaction products trapped inside the sol-gel particles. The sol-gel CW 20M sorbent was synthesized using two catalysts, an acid catalyst during hydrolysis and a base catalyst during polycondensation. Due to the use of two catalysts in the sol-gel synthesis, the CW 20M polymer becomes chemically integrated into the sol-gel network and was able to endure the high temperature without converting into CO_2_ during the thermogravimetric analysis. It should also be noted that the stoichiometric carbon loading of sol-gel CW 20M was approximately 35% compared to 9–10% carbon loading in commercial C18 sorbents. The total weight loss at the end of the experiment was 6.99%. All these findings are reported in Figure 3.

### 2.4. Enrichment Factors Determination

In Table 4 we reported the enrichment factors observed for the tested compounds. As highlighted within the SPE format, Oasis HLB shows the highest values (up to 607-folds), even if sol-gel CW 20M shows more reproducible values for all compounds, especially for cortisone. Contrary to the speculation made in Section 3.6, sol-gel CW 20M did not provide superior extraction performance compared to its commercial counterparts including C18 and HLB sorbents. The unexpected low performance can be attributed to the insufficient cleaning of the sol-gel CW 20M sorbent after the synthesis and conditioning (the TGA profile of sol-gel CW 20M corroborates this hypothesis). As such, the matrix still contains trapped solvent and unreacted reaction byproducts. Therefore, interaction between the target analytes and the sorbent occurred preferentially on the outer surface. Further experimentation is needed to evaluate actual extraction performance of the new sorbent after proper cleaning to ensure that all the trapped solvent and the unreacted reaction byproducts are exhaustively removed.

In Table 4 we also reported the enrichment factors observed for sol-gel CW 20M in the FPSE format. These values, in accordance with previously observed ones [6], were lower than the sol-gel CW 20M in SPE format, even if always comparable (or higher) in respect to other SPE commercially available devices.

It should be noted that, even if the enrichment factors calculated for both the SPE and the FPSE (Table 4) can vary a lot, and for the latter they were always lower than the SPE, the greatest advantage of the FPSE technique lies in the fact that it allows extraction of the analytes directly from the matrix of interest without having to resort to preliminary procedures of protein precipitation and/or cleaning up [4,6,7] to avoid the clogging phenomena.

## 3. Material and Methods

### 3.1. Chemicals, Solvents and Devices

Ciprofloxacin, methyl-*p*-hydroxybenzoate (methyl paraben, IS), sulfasalazine, and cortisone (>98% purity grade), sodium phosphate monobasic and sodium phosphate dibasic (>99% purity grade) and phosphoric acid were purchased from Sigma-Aldrich (Milan, Italy). Acetonitrile (ACN) and methanol (HPLC-grade), DMSO and HCl 37% (RPE-ISO for analysis) were purchased from Carlo Erba (Milan, Italy) and were used without further purification. The water (18.2 MΩ·cm at 25 °C) for HPLC analysis was generated by a Millipore Milli-Q Plus water treatment system (Millipore Bedford Corp, Bedford, MA, USA). 

Solid phase extraction devices evaluated in this work were Oasis HLB (Waters, Milford, MA, USA), Sep-Pak Vac C18 (Waters, Milford, MA, USA), Avantor C18 loaded SPE (J.T. Baker, Avantor Performance Materials, LLC., Center Valley, PA, USA), and novel Carbowax 20M (sol-gel CW 20M, highly polar sorbent possessing poly(ethylene glycol), H[OCH(CH_3_)CH_2_]_n_OH as the building block). All devices were in the format of 1 mL, 30 mg. GraphPad Prism v.4 (GraphPad Software Inc, San Diego, CA, USA) was used for the statistical analysis of experimental data.

### 3.2. Stock Solution and Quality Control Samples

The stock solutions of chemical standards were made at the concentration of 1 mg/mL in the appropriate solvent, referring to solubility tests: ciprofloxacin was solubilized in a mixture MeOH: HCl (0.1 M) 1:1 (*v*/*v*), sulfasalazine in DMSO, methyl-*p*-hydroxybenzoate and cortisone in MeOH. The combined working solutions (at the concentration of 10 and 50 μg/mL) were prepared by dilution of a mixed solution in MilliQ water. The resulting samples were used to evaluate the enrichment factors, breakthrough volume, retention volume, hold-up volume, retention factor, and theoretical plate number.

### 3.3. Apparatus and Chromatographic Conditions

Analyses were performed using an HPLC Thermo Fisher Scientific liquid chromatography system (Model: Spectra System P2000) coupled to a photodiode array detector (PDA) Model: Spectra System UV6000LP. Mobile phase was directly on-line degassed by using a Spectra System SCM1000 (Thermo Fisher Scientific, Waltham, MA, USA). Excalibur v.2.0 Software (Thermo Fisher Scientific, Waltham, MA, USA) was used to collect and analyze the data. The Luna C18 (250 × 4.6 mm, 5 μm particle size; Phenomenex, Torrance, CA, USA) packing column connected to a security guard column (4.0 × 3.0 mm, 5 μm particle size; Phenomenex, Torrance, CA, USA) were used to separate drugs and internal standard (IS). The column and security guard column were thermostated at 25 °C (± 1 °C) using a Jetstream2 Plus column oven during the analysis. Drugs and IS were detected at the maximum wavelengths of 283 nm (ciprofloxacin), 369 nm (sulfasalazine), 260 nm (methyl-*p*-hydroxybenzoate), 247 nm (cortisone), respectively, following the validated method reported in reference [6].

### 3.4. Preparation of Fabric Phase Sorptive Extraction (FPSE) Media

The preparation of the cellulose fabric substrate for sol-gel coating, the preparation of the sol solution for sol-gel coating, and the sol-gel immersion coating process have been described in detail elsewhere [15].

### 3.5. Synthesis of Sol-Gel CW 20M SPE Sorbent

The sol solution for sol-gel CW 20M SPE sorbent was prepared by mixing Carbowax 20M polymer, tetramethoxysilane (TMOS), methyl trimethoxysilane (MTMS), and isopropanol in a molar ratio 0.015:1:1:20, respectively, as reported by Kabir and Furton [17]. The mixture was first vortexed for 5 min and subsequently sonicated for 30 min to ensure that the resulting solution became homogeneous and free of any trapped bubbles. Afterwards, 0.1 M HCl was added to the mixture in a molar ratio of TMOS: 0.1 M HCl, 1:4. The mixture was mixed again for 3 min and the sol solution was left overnight thus that the hydrolysis of the sol-gel precursors, TMOS and MTMS, continued towards completion. Subsequently, 1 M NH_4_OH was added in droplets to the sol solution under vigorous stirring until the pH of the solution became basic and the sol solution started turning into a gel spontaneously. Once the gelation completes, the sol-gel matrix was allowed to age for 48 h. The sol-gel matrix was then crushed and dried in a vacuum oven at 70 °C for 48 h. The dried sol-gel CW 20M SPE sorbent was then rinsed with 50:50 (*v*/*v*) methanol: Methylene chloride under sonication to remove unreacted sol solution ingredients and reaction byproducts. The sol-gel CW 20M sorbent was dried again at 70 °C for 24 h. Finally, the dried sol-gel CW 20M sorbent was pulverized in a ball mill into fine particles and particles between 40–52 µm diameter were collected in a mesh screen for using as the SPE sorbent.

### 3.6. Preparation of the SPE Media

The SPE cartridges used in this work were both commercial (Sep-Pak Vac C18, Oasis HLB, and Avantor C18) and in-house loaded with the sol-gel CW 20M (this latter stationary phase was synthetized as previously reported in paragraph 3.5). All devices were obtained in the format of 1 mL, 30 mg in order to compare the analytical performances directly. The principal SPE phase characteristics are reported in Table 5.

Compared to Brunauer–Emmett–Teller (BET) adsorption isotherm values (surface area, pore diameter, and pore volume) obtained for sol-gel CW 20M SPE sorbent with commercially available HLB and C18 sorbents, sol-gel CW 20M SPE sorbent demonstrated higher surface area and pore volume and is expected to provide superior extraction efficiency compared to their commercial counterparts. 

### 3.7. SPE Sorbents Characterization Using FTIR and TGA

FTIR spectroscopy was conducted on a Perkin-Elmer spectrum TWO instrument, operating in Attenuated Total Reflectance (ATR) mode in the 4000 cm^−1^–450 cm^−1^ wavenumber range. Thermogravimetric analysis was carried out using a Netzsch Regulus 2500 thermobalance, in air atmosphere, in the 25–950 °C temperature range.

## 4. Conclusions

In these researches, it was highlighted that for the tested compounds nowadays there is not available a “universal” phase. In fact, for some analytes it was not possible to evaluate the breakthrough volume (*V_B_*), retention volume (*V_R_*), hold-up volume (*V_M_*), retention factor (*k*), and theoretical plates number (*N*), because no quantitative data were obtained after the SPE steps. Consequently, it was not possible to perform the analysis by Boltzmann’s function due to a “floating point error”. 

Comparing the breakthrough volume and enrichment factors data allowed us to evaluate the performance of both the FPSE and SPE techniques. The SPE technique showed the highest enrichment factors; consequently, this method is more suitable for samples with low analytes concentration. Indeed, breakthrough volumes and the number of theoretical plates data from the SPE method are higher for analytes concentration at 10 µg/mL, than the analytes concentration at 50 µg/mL. Generally, the deployment of silica sorbents (e.g., Avantor C18 and Sep-Pac-Vac C18) is not that advantageous relative to polymeric sorbents (e.g., Oasis HLB and sol-gel CW 20M) due to their instability in broader pH ranges. Additionally, silanol groups in silica sorbents bind irreversibly with target components, thus requiring more eluting solvents and a large consumption of time [18,19]. 

Among the different sorbent phases tested, Oasis HLB cartridges have the best performances, furthermore, against expectations, we found that even C18 cartridges showed better results than sol-gel CW 20M cartridge for that kind of analytes. 

## Figures and Tables

**Table 1:** Molecular structures and other pertinent physiochemical properties of selected inflammatory bowel disease (IBD) drugs and the internal standard.

Drug Name	CAS No.	Molecular Weight (g/mole)	Molecular Structure	LogP	pK_a_
Cortisone	53-06-5	360.44	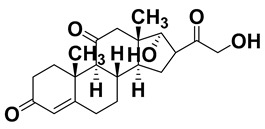	1.66	12.6
Methyl-*p*-hydroxy benzoate	99-76-3	152.15	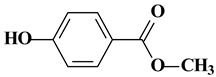	1.96	8.4
Ciprofloxacin	85721-33-1	331.34	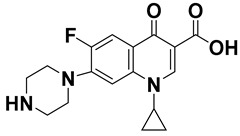	2.30	5.76
Sulfasalazine	599-79-1	398.39	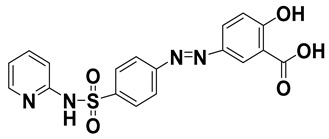	2.92	3.3

**Table 2:** Parameters determined for the analytes on different sorbents in frontal analysis at the concentration of 10 µg/mL.

Sorbent	Analyte	Breakthrough Volume (mL)	Retention Volume (mL)	Hold-Up Volume (mL)	Retention Factor (*k*)	Number of Theoretical Plates (*N*)
Avantor C_18_	Ciprofloxacin	>50	-	-	-	-
	Sulfasalazine	>50	-	-	-	-
	Methyl-*p*-hydroxybenzoate	11.3	9.9	11.9	0.20	2000
	Cortisone	>50	-	-	-	-
Sep-Pac Vac C_18_	Ciprofloxacin	23.6	16.4	30.6	0.87	16
	Sulfasalazine	>50	-	-	-	-
	Methyl-*p*-hydroxybenzoate	52.8	37.3	66.8	0.79	18
	Cortisone	>50	-	-	-	-
Oasis HLB	Ciprofloxacin	27.6	25.9	28.8	0.11	845
	Sulfasalazine	>50	-	-	-	-
	Methyl-*p*-hydroxybenzoate	>50	-	-	-	-
	Cortisone	>50	-	-	-	-
CW 20M	Ciprofloxacin	5.26	2.2	8.7	2.95	1
	Sulfasalazine	>50	-	-	-	-
	Methyl-*p*-hydroxybenzoate	4.4	2.9	4.9	0.66	13
	Cortisone	6.5	4.5	7.9	0.75	17

**Table 3:** Parameters determined for the analytes on different sorbents in frontal analysis at the concentration of 50 µg/mL.

Sorbent	Analyte	Breakthrough Volume (mL)	Retention Volume (mL)	Hold-Up Volume (mL)	Retention Factor (*k*)	Number of Theoretical Plates (*N*)
Avantor C18	Ciprofloxacin	15.1	8.8	32.2	2.68	5
	Sulfasalazine	29.7	17.4	46.5	1.67	5
	Methyl-*p*-hydroxybenzoate	6.7	3.7	14.3	2.91	3
	Cortisone	25.7	14.2	49.6	2.48	4
Sep-Pac Vac C18	Ciprofloxacin	5.4	3.6	7.2	0.99	12
	Sulfasalazine	>20	-	-	-	-
	Methyl-*p*-hydroxybenzoate	21.4	14.7	27.4	0.87	15
	Cortisone	>20	-	-	-	-
Oasis HLB	Ciprofloxacin	13.9	8.8	20.7	1.34	8
	Sulfasalazine	>20	-	-	-	-
	Methyl-*p*-hydroxybenzoate	>20	-	-	-	-
	Cortisone	>20	-	-	-	-
CW 20M	Ciprofloxacin	>20	-	-	-	-
	Sulfasalazine	13.2	7.5	22.7	2.02	4
	Methyl-*p*-hydroxybenzoate	>20	-	-	-	-
	Cortisone	>20	-	-	-	-

**Table 4:** Enrichment factors (%) observed at concentrations of 0.8, 2.5, and 8 µg/mL of water standard solutions. The enrichment factors were calculated as the percentage of peak area enhancement (after extraction) with respect to the area of reference standard solutions.

Analyte	QC Concentration (μg/mL)	FPSE CW 20M	SPE Format			
			Avantor C18	Sep-Pac Vac C18	Oasis HLB	CW 20M
Ciprofloxacin	0.8	28.4	**740**	304	67	321
	2.5	37.0	406	199	**607**	186
	8	13.6	200	188	**367**	113
Sulfasalazine	0.8	248	**640**	100	380	202
	2.5	164	325	206	**408**	217
	8	278	344	299	**493**	225
Methyl-*p*-hydroxybenzoate	0.8	190	204	78	**314**	59
	2.5	155	310	133	**344**	113
	8	121	205	216	**252**	76
Cortisone	0.8	136	80	123	n.a.	**171**
	2.5	88	160	195	167	**199**
	8	128	203	240	**245**	161

n.a. not available, the analyte is not detected during the analysis.

**Table 5:** Physical characteristics of SPE sorbent phases.

Sorbent	Sorbent Mass (mg)	Surface Area (m^2^/g)	Pore Diameter (Ǻ)	Pore Volume (cm^3^/g)	Particle Size (µm)
Oasis HLB	30	800	80	1–3	30
Sep-Pac Vac C_18_	30	325	125	-	55–105
Avantor C_18_	30 *	320–350	60	-	40
Carbowax 20M	30	990	71	1.8	40

* Commercially available in the size of 1 mL/100 mg sorbent phase; to compare cartridges with the same amount of sorbent phase, we have emptied and reconstituted them with 30 mg of their sorbent phase.
